# Hints to Consumptives

**Published:** 1860-12

**Authors:** F. W. Hemming

**Affiliations:** Savannah, Ga.


### ARTICLE III.

*Hints to Consumptives.* By F. W. HEMMING, M. D., Savannah, Ga.

1. Be sure you have consumption, or, from hereditary causes are likely to prove its victim, before you assume the serious responsibility of combating within you so formidable a disease that has not attacked you yet, and may never. To ascertain this, employ a competent physician, a man of honor and character as well as capability; whatever may be the condition of your lungs such an one *can* and will inform you. If you must do so, travel far at any expense to find a man so distinguished. You can form no idea of the ravages to be made on your purse and property by the incompetent and dishonorable, if you neglect your duty, and sacrifice health and life at the bidding of the ignorant and unprincipled. Auscultation reveals only to the educated ear the secrets that are hidden from the eye beneath the breast. The foliage of the lofty oak is spread abroad in the face of heaven to bring light and vitality to its stalwart frame. The foliage of man is condensed into a wonderful texture that nestles around the precious heart, and brings light and vitality to that fountain of life, hidden deep within his bosom, sending the water of life by a million streams to nurture every part of his wonderful but delicate structure. As the wind murmurs through the leaves we know not its language, and its voice is unheeded; but its faintest whisper in the lungs is like the still small voice of the Almighty, whose merciful warnings we dare not disregard—whose solemn but truthful oracles abash the soul in awe. The ocean roars, and rocked upon its billows, we tremble at its fury, are appalled by its rude bellowings; the rippling brook charms by its melody; yet neither ocean nor streamlet utters tones we understand, but each sound that comes gurgling from the heart is either of fearful importance or of cheering interest; we know every syllable, alas, too well. The practised auscultatist alone can assure you of the probability of your longevity. The judge may condemn the culprit to death, but a merciful, if not erring, executive may prolong the sentence. When the practised auscultatist pronounces sentence upon you, your doom is inevitable; you cannot escape it. My reader, why should you go to the fool to be like him in his folly, passing by the door of the minister of health, which God gives only to intelligent and grateful recipients. Neglect him if you like, trifle with your health if you dare, you alone incur the guilt of the suicide; he is clear of your blood. You go to your grave; you have enriched the imposter who laughed at your credulity, and mourned sincerely the loss of your purse. Who is most guilty, the knave, or the supporter of knavery? You will be wise if you answer that question on this side of the grave.

2. It will prove no strange thing if you find yourself assured by competent authority that you or your children may, ere long, become the victims of consumptions, although the disease is either dormant, or has even given no signs of its presence. Of all who die, whose deaths are recorded, one in eight die of consumption. In most cities the deaths from consumption are one in five. In others, one in four. In Paris and London, one in three. Of all the youthful who die between the periods of birth and puberty, one in four die of consumption; and in the female sex the proportion is much greater—one in three does not fairly represent it. In the United States and Great Britain together, the deaths from consumption are about three hundred daily, or as every five minutes have passed, another victim has been slain. If you value life, and would have your children spared, the solemn responsibilities resting upon you must be fulfilled. You have forgotten, perhaps, that the first business of life is to live. Is this all? Have you learned how to live? And will your children learn from you how to live—usefully, happily and holy? Death comes not unbidden to those who refuse to learn these lessons.

3. What can be done to prevent consumption, you ask? Doctors cannot prevent it, medicines cannot, all that can be done you must do, and if success is possible it is almost certain to crown your efforts. Success may be impossible if the constitution is so depraved or enfeebled from certain causes that an early death becomes an inevitable certainty; yet, otherwise, all things being equal, success is almost certain. There are certain habits in life that must be practised no longer, peculiar conditions which must be avoided, imperative necessities which must be supplied, and the simplest of duties scrupulously performed, that are daily disregarded, though of daily importance. Hereditary weakness of constitution, or proneness to disease on your part, is a misfortune calling for sympathy and alleviation. To transmit these to posterity without attempting to eradicate them, or modify their influences, is guilt as well as folly.

4. Are there no habits to be rectified? *First*, Uncleanliness of person. This is a grave charge, not lightly alleged. Consumptives habitually disregard cleanliness. Their clothing and bedding reek with offensiveness; they shrink from water as if mad with hydrophobia. They are not alone in this: The *mighty unwashed* give the hands two or three hasty rubs, the face is smeared over once or twice, eyes and ears are conscientiously untouched, and their ablutions are finished. Others go a little farther: soap and water for the hands, face and neck, sometimes to the feet, but they sacredly cherish every unseen spot, and preserve it from the touch of moisture. The face and hands return largely this grudged devotion. Unclad and unburdened, they brace every extreme of heat and cold, whilst the debilitated body, begrimmed and covered, is affected deeply by every change of temperature. A bath-room is of more importance than a parlor. Its absence is no excuse for filthiness. Every morning one quart of soaped water will be sufficient to cleanse the person, if water is scarce in your vicinity. A towel dipped in this water and applied to different parts of the body in succession, will be all that is necessary. First wash a part, then dry it thoroughly, keeping the rest of the body carefully covered, and no harm can ensue to the feeblest constitution, even in the coldest climate. Let the water be as cold as can be tolerated with comfort (tepid will do), use moderate friction, follow it up with gentle exercise, and you will experience throughout the day an enjoyment that has been cheaply purchased, and receive a benefit, the worth of which you can never properly estimate. Use *warmer* water the same way in the evening on retiring. Your sleep will be sound and refreshing, and in the morning, with greater benefit than before, you will experience the result of your morning's ablution. The strong may neglect this, perhaps, with impunity. The debilitated do so at the peril of life. *Second*, Constant use of medicine. Never take any medicine unless sanctioned by grave circumstances, as prescribed by conscientious, intelligent physicians. The use of those pernicious compounds known as patent medicines, however efficacious under certain circumstances, inflicts, necessarily, irremedial injury on their consumers. You believe the roll of questionable affidavits, but not with sufficient faith. Were they true, not a vestige of disease would abound. You would have been cured long ago. Can you not distinguish between the claims of those who vaunt their *own* pretensions, and those whose worth is estimated by a discerning community? The constant use of any one medicine as certainly predisposes the body to disease, as the use of any one article of food to the exclusion of others will as certainly hasten an untimely death. *Third*, Suppression of the evacuations. This disgusting habit is not the fault of the body, but of you. A proper diet of a mixed character, with prompt attention to every call of nature, will obviate this error. Stewed or ripe fruits eaten at breakfast, animal food with a moderate amount of fat or olive oil at dinner, good, sweet butter eaten with cold home-made bread, and stewed fruit for supper, will as rapidly remove this difficulty as a farinaceous diet will correct, naturally, a relaxed condition. Let your water-closet be well washed with water after using; let no effluvia ever taint your house; use disinfectants if necessary. The out-houses of the day are a disgrace to the age—terrible aids to the epidemics that would be less terrible without them. The fluid should be voided from the body at least thrice a day, and the solids invariably once. The subject is too repulsive to tell you the results of retaining them within you day after day. Yet how often is this done; but never without injury. Use no laxatives but what your physician considers best adapted to you. *Fourth*, Depressing passions. A grateful sense of the boundless mercies you enjoy, proper employment, cheerful association with the pure and intelligent, the perusal of *good* books, and healthful recreation, will banish them under God's blessing from your heart and home; but above all, the enjoyment of a religion of love, exercised by holy charity, to all, especially to the needy, sick, and unfortunate, will dispel the last shade of gloom. Religion is being good, and doing good. Live to act. *Fifth*, Intemperance. Only the moderate use of wholesome articles is tolerated by nature. He who tampers with the unwholesome has to combat an enemy who has slain his thousands. *Sixth*, Sedentary habits. Contraction of the chest and deficient aeration of the blood, are the dreadful penalties that threaten the consumptive in a life of sedentary habits, but loss of appetite, impaired digestion, want of sleep, an enfeebled constitution, depressing passions, intemperance, and insanity too often follow as the melancholy results. *Seventh*, Excessive exertion of mind and body. Mind is not developed by forcing. The cruel practice of immuring children six hours in a school-room, to be followed by more hours of severe study at home to get their lessons for the morrow, cannot be too strongly reprobated. I have seen children poring over their tasks at ten o'clock at night, urged on by their thoughtless parents to a precocity as fatal as it was useless. This is nothing less than a refinement of infanticide. Children should learn by the eye the nature of the objects around them; be taught orally those facts they are so anxious to learn by persistent enquiry; have the physical system carefully prepared to endure the study of those more difficult attainments requiring application; and no attainment should be made at the expense of health. Excessive exertion of the body is seldom the cause of consumption, yet it has its influences on the robust. Men of vigor become victims to consumption by their eager, restless and insatiable pursuit of wealth, imprisoned in stores, counting-houses, and manufactories, strangers to their families, whom they seldom see but at night or on Sabbath days (the family circle being a fact, but composed of links too wide apart), the wealth is obtained sometimes, and secures a splendid mausoleum for the parent, leaving his orphans as a prey to the designing. Too many of our young men sink their manhood in the most effeminate employments. Selling tape and buttons may be proper to a delicate female, but a man can find nobler pursuits in scientific agriculture, mechanics, or the fabrication of articles requiring manly strength, vigor, and activity.

5. The peculiar conditions to be avoided are, briefly: *First*, Improper occupations. If your occupation is too confining, or impairs your health, you must change it, or devote only so much time to it that the remainder may be spent in renewing your strength. Wealth without health is a poor substitute for health without wealth. *Second*, Unsuitable climates. Consumption is a disease of temperate latitudes—not of the colder or warmer climes. The latter are the safest. A residence, moderately elevated above the level of the sea, can be secured anywhere in the South, at a trifling cost, where all the necessaries of life can be obtained abundantly and cheaply by those pursuing any proper occupation. *Third*, Bad habitations. A poor house, in any climate, will be no refuge for you. A residence in a crowded city, or in a consumptive hospital, anywhere, is as foolish as pernicious. A home in some rural, quiet spot, free from epidemics, well-ventilated, drained and supplied with good water, is a home that may be a paradise if you will it.

6. The necessities of life and health are easily supplied; a few words will comprise them: Pure air, freedom from effluvia, constant bathing, proper clothing, and good nourishment. *First*, Proper clothing. The softest flannels that a babe should wear should clothe the entire person in summer, next to the skin, and warmer and heavier kinds in winter. Take them off at night, even should you sleep in a flannel gown. These, with good shoes (with movable cork soles for damp weather), and woollen stockings, are the essentials of your wardrobe; the other articles, as long as they do not confine the chest or abdomen, wear, if you like, as fashion dictates. *Second*, Good nourishment. Not what you eat will nourish you, but what you digest. The kind of food has been already indicated. Earn your appetite as well as your meal.

7. Your daily duties then, as regards health, will be: Exercise of the lungs and body in the open air, temperance in all things, nourishing diet, fairly earned—not the bread of idleness or dependence; cleanliness of person, and last but not least, physical development. You have to fight for health as well as strength. The brutal prize-fighter accomplishes the development of his person only to bear its bruising and battering in a disgraceful combat. The athlete never acquired his manly form and Herculean powers but by conforming to the laws of health and nature. Something less than the strength of either would make life a blessing to you instead of a burden. But you will have to exercise patience and self-denial for nobler purposes. This is not a matter of experiment but of fact. Many examples might be given. Let the following suffice: In Great Baitain consumption is rife; the deaths amongst children, out of all diseases, being one in four. In certain public schools, whose statistics are well known to the writer, there were twelve hundred children instructed and fed during eight years. The deaths per annum were less than one per cent. Not one death occurred from consumption during this period. The diet was a breakfast of bread and milk, a dinner of meat, bread, vegetables, and water, and a supper of bread and cheese, and water. Bathing, or rather washing of the person in tepid water every evening, and every morning in colder watei, was rigorously exacted. The hours for study were six in winter, and seven in summer. Vigorous play was enjoyed for four hours on every week-day, besides the weekly holidays. The children's ages were from seven to seventeen. The puny children who entered these institusions rapidly became strong and healthy, and acquired habits of temperance, frugality, and hygiene, that will be their heritage through life.

